# Actinomycose pelvienne pseudo tumorale associée au dispositif intra-utérin: à propos de trois cas

**DOI:** 10.11604/pamj.2014.19.87.4747

**Published:** 2014-09-26

**Authors:** Moulay Elmehdi El Hassani, Abdellah Babahabib, Jaouad Kouach, Farid Kassidi, Younes El Houari, Driss Moussaoui, Mohamed Dehayni

**Affiliations:** 1 Service de Gynécologie Obstétrique, Hôpital Beaujon, AP-HP, Paris, France; 2 Service Gynécologie Obstétrique, Hôpital Militaire d'Instruction Mohamed V, Rabat, Maroc

**Keywords:** Actinomycose pelvienne, pseudotumeur, dispositif intra-utérin, pelvic actinomycosis, pseudotumor, IUD

## Abstract

L'actinomycose est une maladie chronique suppurative granulomateuse d'origine infectieuse responsable d'un syndrome tumoral. La localisation pelvienne est rare et souvent associée, chez la femme, au port au long court du dispositif intra-utérin (DIU). Le diagnostic préopératoire n'est possible que dans 17% des cas. Nous rapportons trois observations, d'actinomycose pelvienne pseudo tumorale compliquées chez des femmes porteuses de DIU, qui illustrent le rôle de ce moyen de contraception dans la genèse de cette pathologie ainsi que les difficultés de prise en charge.

## Introduction

L'actinomycose pelvienne est une maladie suppurative rare, due à une infection par un bacille Gram positif anaérobie du genre Actinomyces [1]. L'actinomyces israelii est l'espèce le plus rencontré en pathologie humaine, Il est considéré comme une bactérie commensale occasionnelle de la flore vaginale et peut devenir pathogène en cas d'effraction muqueuse [1, 2]. L'actinomycose pelvienne chez la femme est rare et souvent associée au port d'un dispositif intra-utérin (DIU) [3] et peut revêtir une forme pseudo tumorale. Son incidence est en augmentation du fait de l'usage de plus en plus fréquent de stérilet comme moyen de contraception. Le diagnostic est difficile, il est souvent histologique, en effet le tableau clinique est peu spécifique. A travers trois cas d'actinomycose pelvienne pseudo tumorale chez trois patientes porteuses de dispositif intra-utérin (DIU), nous essayons de montrer le rôle du DIU dans la genèse de cette pathologie et d'en rapporter les difficultés diagnostiques, les modalités évolutives et les possibilités thérapeutiques.

## Patient et observation

### Observation N°1

**Figure 1:**
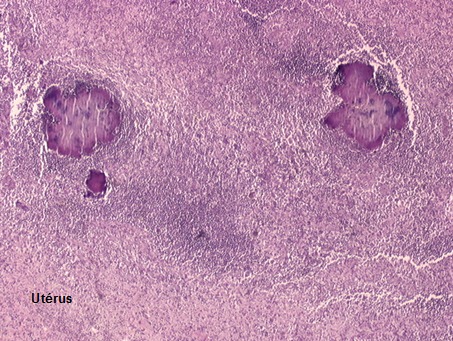
Grains d'actinomycose; petites masses amorphes basophiles éparses dans un granulome inflammatoire d'apparence non spécifique, elles sont formées d'un feutrage de filaments représentant l'agent pathogène

Mme G.A, âgée de 45 ans, primigeste, primipare, sans antécédents pathologiques particuliers, porteuse d'un DIU depuis 5 ans était hospitalisée pour une altération de l’état général. Le début de la symptomatologie remontait à 6 mois par des douleurs abdomino-pelviennes associée à une diarrhée, fécalurie et hématurie, le tout évoluait dans un contexte fébrile d′anorexie et d'amaigrissement chiffré à 13Kg. La patiente était cachectique, fébrile à 38,5°c et la palpation abdominale trouvait une masse au niveau du flanc droit ferme et irrégulière. Le toucher vaginal (TV) trouvait un pelvis blindé, le DIU est retiré sous spéculum. Le reste de l'examen avait montré un oedème du membre inférieur droit d'aspect inflammatoire. L’échographie et le scanner abdomino-pelvien mettaient en évidence deux masses pelviennes et des images hépatiques multiples évocatrices soit de métastases soit d'abcès, il existait également une dilatation des cavités pyélo-calicielles surtout à gauche et une phlébite ilio-fémorale droit. Il n'y avait pas d'ascite ni de carcinose péritonéale. La radiographie pulmonaire était normale. Les examens biologiques révélaient un syndrome inflammatoire avec une protéine C réactive (CRP) à 175 mg/l. L′hémogramme trouvait un taux de globules blancs à 26000/mm^3^ à prédominances polynucléaires neutrophiles, Une anémie à 8,2 g/dl hypochrome microcytaire, un taux de plaquettes à 180000/mm^3^. La fonction rénale trouvait une créatinine à 55micromol/l. Devant l'altération de l’état général, les multiples images d'abcès et surtout la présence d'un DIU négligé depuis 5 ans, le diagnostic d'actinomycose pelvienne était évoqué en premier ou un cancer de l'ovaire avec métastases hépatiques. La laparotomie exploratrice avait montré deux gros ovaires, un abcès pelvien communiquant avec la vessie et le rectum, un abcès du flanc droit et cinq abcès hépatiques superficiels. Une ovariectomie bilatérale avec hystérectomie subtotale était réalisée; l'examen extemporané de l'ovaire gauche avait objectivé une actinomycose et éliminer une néoplasie; le geste était complété par un drainage des différents abcès, une suture des fistules vésicale et rectale et terminé par une colostomie gauche. L'examen anatomopathologique définitif concluait à une actinomycose multifocale sans lésion tumorale associée (Figure 1). La patiente a été mise sous anticoagulant et une pénicillothérapie G 18 millions/jour en IV en trois prises pendant six semaines relayée par l'amoxicilline per os 6 g/j pendant 9 mois. Ce traitement a permis une amélioration de l’état général avec correction de la fonction rénale, disparition des différentes lésions au scanner et à l’échographie et une régression de la phlébite au bout des trois premier mois. Le rétablissement de la continuité fut réalisé à 6 mois avec succès. La patiente est restée en rémission clinique et biologique avec un recul de 5 ans.

### Observation N°2

Patiente âgée de 52 ans, dixième geste deuxième part ménopausée depuis un an, porteuse d'un DIU depuis 7 ans. Hospitalisée en urgence pour syndrome subocclusif dans un contexte d'altération de l’état général avec perte de 6 kg en 3 mois. L'examen gynécologique trouvait un blindage pelvien, un col d'aspect sain et le fil de stérilet n’était pas vu, les culs de sac étaient fixés, le rectum et la vessie infiltrés. L’échographie pelvienne et le scanner abdomino-pelvien trouvaient deux images kystiques ovariennes hétérogènes associées à une infiltration du sigmoïde et du rectum haut d'aspect tumoral avec dilatation modérée des deux uretères, le DIU était en place. Biologiquement, la vitesse de sédimentation (VS) était à 100mm, la CRP était à 175mg/l, les globules blancs à 18000/mm^3^; Les marqueurs tumoraux sériques étaient négatifs. ACE: 0,8ng/l (N< 37) et CA125: 52 (NFigure 2). La patiente était traitée par Pénicilline G 18 millions d'unités par jour pendant 6 semaines puis relais par Pénicilline V 4 millions 4cp par jour pendant un an. L’évolution immédiate fut marquée par une amélioration de l’état général avec correction des troubles biologiques, ablation des sondes double J à 3 mois avec fonction rénale et diurèse normales et rétablissement de la continuité digestive à 6 mois avec succès. La patiente était asymptomatique avec un recul de 6 ans.

**Figure 2:**
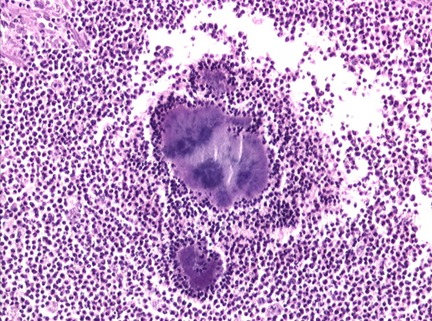
Granulome inflammatoire d'actinomycose

### Observation N°3

**Figure 3:**
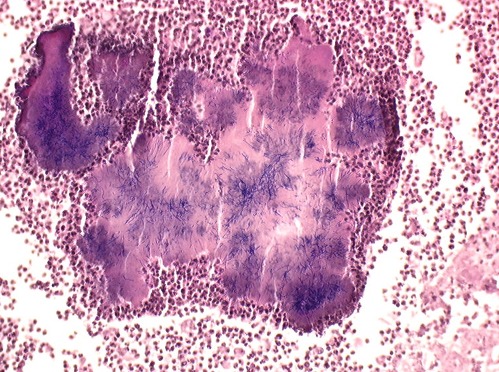
Aspect filamenteux d'actinomycète

Une femme de 55 ans quatrième geste quatrième part. Diabétique sous insuline, porteuse d'un DIU oublié depuis 15 ans. Admise pour pelvipéritonite avec syndrome tumoral pelvien. L'examen trouvait une défense pelvienne, un col sain, le fil de stérilet n’était pas vu au spéculum et au toucher vaginal les culs de sac étaient fixés. Biologiquement les globules blancs étaient à 26000/mm^3^, la CRP à 270mg/l, l'hémoglobine à 10 g/dl, la glycémie à 2,10 g/l et la fonction rénale était normale. L’échographie et la TDM abdominopelvienne montraient deux images kystiques hétérogènes de part et d'autre de l'utérus dont la plus grande mesurait 6cm. Une laparotomie trouvait un pelvis très adhérentiel, l'adhésiolyse laborieuse avait permis de découvrir un pyosalpinx bilatéral avec aspect cartonné de la face postérieure de la vessie. Une hystérectomie totale avec annexéctomie bilatérale et des biopsies de la face postérieure de la vessie furent réalisées. L'examen extemporané avait éliminé une pathologie néoplasique. L’étude anatomopathologique définitive a conclu à une actinomycose au niveau des annexes et de l'utérus avec aspect filamenteux d'actinomyces (Figure 3). La patiente fut mise sous Pénicilline G 18 millions/jour pendant 4 semaines suivie de 9 mois d'amoxicilline à raison de 6 g/jour. L’évolution immédiate fut favorable et le suivi régulier en consultation montrait une patiente en bon état général sans signe de récidive avec un recul de 5ans.

## Discussion

L'actinomycose est une maladie infectieuse rare, l'agent causal est l'actinomyces israelii, décrit pour la première fois par israel en 1978 [2]. Il s'agit d'une bactérie commensale à gram positif, filamenteuse, ramifiée et anaérobie [2]. Les localisations les plus fréquentes sont cervico-faciale (50-60%) et thoracique (20-25%) [2, 4]. L'actinomycose abdominopelvienne est rare mais sa prévalence augmente avec l'utilisation croissante des stérilets [5, 6]; en effet, le germe est identifié chez 8-20% des femmes porteuses de DIU [7] et le premier cas d'actinomycose abdomino-pelvienne associée à un DIU a été rapporter par Henderson en 1973 [3]. Le DIU provoquerait un traumatisme prolongé de l'endomètre, induisant des zones de nécrose en présence d'une pathologie inflammatoire pelvienne préexistante, réalisant un environnement favorable au développement de l'actinomycose [8]. Le pouvoir pathogène d'actinomyces est favorisé par la présence d'autres germes (E. coli, streptocoque, flore anaérobie) [4] et la sévérité de l'infection dépondrait de la quantité d'enzymes protéolytiques sécrétées par le germe et la qualité de la réponse immunitaire de l'hôte. Ce qui explique la variabilité clinique qui va du portage chronique aux formes pseudo tumorales chroniques et parfois des formes aigues péritonéales. Le diagnostic préopératoire de l'actinomycose demeure très difficile, en effet, seule 17% d'actinomycose pelvienne sont diagnostiquées en préopératoire [9]. Néanmoins, il est indispensable car sa méconnaissance risque de pousser le chirurgien à des actes chirurgicaux radicaux, morbides et mutilants surtout chez la femme jeune. Le syndrome tumoral est souvent au premier plan avec une masse pelvienne mal limitée, fixée, et douloureuse; associée à une altération de l’état général parfois cachexie avec un état fébrile. Le syndrome inflammatoire biologique est constant et marqué. Le taux de CA 125 est parfois augmenté [10]. L'extension aux organes de voisinage est possible notamment vers la vessie et le recto sigmoïde avec tendance à la fistulisation. L'atteinte retro péritonéale est fréquente avec compression urétérale et/ou vasculaire avec parfois hydronéphrose ou thrombophlébite (c'est le cas de notre première patiente) et même atteinte vertébrale [11]. L'imagerie (échographie, scanner ou résonnance magnétique nucléaire) confirme le syndrome tumoral, montre son extension mais ne permet pas de faire le diagnostic d'actinomycose. En effet, elle peut montrer des images évoquant des abcès pelviens, un processus tumoral bénin ou malin surtout d'origine ovarienne, un processus inflammatoire ou infectieux; elle permet aussi d’établir un bilan d'extension locorégional [11, 12]. En premier lieu sont évoqués les diagnostics de cancer (colique, ovarien, utérin ou vésical), d'endométriose sévère, de pelvipéritonite, de maladies inflammatoires digestives notamment la maladie de Crohn, de sigmoïdite ou d'appendicite compliquée voire de tuberculose urogénitale ou colo-intestinale.

Le diagnostic d'actinomycose peut être suspecté chez toute femme porteuse de DIU au long court, devant l'association d'un syndrome inflammatoire et d'un syndrome tumoral pelvien. Il n'existe pas de diagnostic sérologique en routine. Le diagnostic préopératoire pourrait être bactériologique, il est cependant difficile à établir. Il est obtenu après ponction d’éventuelles collections sous échographie ou sous scanner. La fragilité du germe et sa culture difficile et longue, rendent souvent cette ponction non contributive au diagnostic [7]. La détection de l'actinomyces par le frottis cervicovaginal dans un contexte particulier doit pousser le praticien à plus d'investigations [5, 13] Généralement, devant le doute diagnostic, une laparotomie exploratrice est réalisée; l'examen extemporané permet d'exclure une néoplasie; et le drainage des abcès. L'examen histologique des pièces opératoires confirme souvent le diagnostic. Il montre typiquement de nombreux abcès constitués de polynucléaires altérés, entourés d'un infiltrat histolymphoplasmocytaire et d'une couronne riche en fibrine et collagène [14, 15]. Au centre de ces abcès, on retrouve les caractéristiques « grains de soufre » ou « grains actinomycosiques » du centre desquels des filaments s’échappent en rayon de roue [14].

Le traitement est à base de pénicilline G à forte dose: 18 à 20 million/jour pendant 4 à 6 semaines avec un relais par voie orale pendant 6 mois à 1 an [16]. En cas d'allergie à la pénicilline on peut utiliser les macrolides, les cyclines ou la rifampicine [7]. Dans l'actinomycose abdominopelvienne pseudo tumorale, l'exérèse complète des tissus infectés est rarement possible. La persistance de foyers non stérilisés expose à un risque de rechute. Le pronostic est généralement favorable sous traitement antibiotique bien suivi. Toutefois, des complications graves par diffusion locale ou généralisation par atteinte viscérale à distance ont été rapportées. Elles justifient une surveillance prolongée [13].

## Conclusion

L'actinomycose pelvienne est une pathologie rare qui doit être évoquée chez toute femme porteuse d'un stérilet depuis plusieurs années et présentant une altération de l’état général, un syndrome inflammatoire et un syndrome tumoral pelvien. La méconnaissance de l'affection peut conduire à un retard diagnostique avec risque de survenue de complications graves. Le diagnostic est souvent histologique. Le traitement est essentiellement médical basé sur une antibiothérapie au long cours.
